# Supplementary material for: Five copper homeostasis gene clusters encode the Cu-efflux resistome of the highly copper-tolerant Methylorubrum extorquens AM1
Source: PeerJ. 2023 Feb 20;11:e14925. doi: 10.7717/peerj.14925 (PMC9948745; doi:10.7717/peerj.14925)
Supplement: Supplemental Information 1 [file peerj-11-14925-s001.docx]

**Row data of minimal inhibitory concentrations of CuCl_2_ (MICs).**

We followed the definition and standardized dilution method set by the British Society of Antimicrobial Chemotherapy and updated by (Andrews, 2001), and by the European Committee for Antimicrobial Susceptibility Testing (EUCAST, 2000) to estimate the MICs of an antimicrobial agent. Based on these studies, the MIC for each strain was defined as the lowest concentration of CuCl_2_ that consistently prevented visible growth in at least three independent assays. Based on this definition, our raw data were images of solid medium with increasing millimolar (mM) concentrations of CuCl_2_ with or without growth of resistant or susceptible strains. Representative examples of growth monitored photographically at 2, 4, and 6 days post-propagation are shown in Fig. S2 (Supplementary material). Numerous images were used to construct a table of permissive and inhibitory concentrations of CuCl_2_ for each strain (supplementary material, Table S2). The workflow used to assess MICs is shown in the supplementary file Workflow for MICs.

**Key factors for maintaining repeatability among MIC assays.**

a) The inoculum must be rigorously prepared as detailed in the material and methods in the different assays.

b) MIC assays must be done in agar plates with chemically defined medium. The use of solid PY rich medium reduced repeatability, it may be due to the high binding affinity of Cu(I) for amino acids.

c) The 50 mM stock solution of CuCl_2_-2H_2_O added to chemically defined medium should be used on the day of preparation and then discarded.

**References**

Andrews, J. M. (2001). Determination of minimum inhibitory concentrations. *Journal of Antimicrobial Chemotherapy*, *48*(suppl_1), 5–16. https://doi.org/10.1093/jac/48.suppl_1.5

EUCAST. (2000). Determination of minimum inhibitory concentrations (MICs) of antibacterial agents by agar dilution. *Clinical Microbiology and Infection*, *6*(9), 509–515. https://doi.org/10.1046/j.1469-0691.2000.00142.x
