# Supplementary figures and images for: Five copper homeostasis gene clusters encode the Cu-efflux resistome of the highly copper-tolerant Methylorubrum extorquens AM1
Source: PeerJ. 2023 Feb 20;11:e14925. doi: 10.7717/peerj.14925 (PMC9948745; doi:10.7717/peerj.14925)

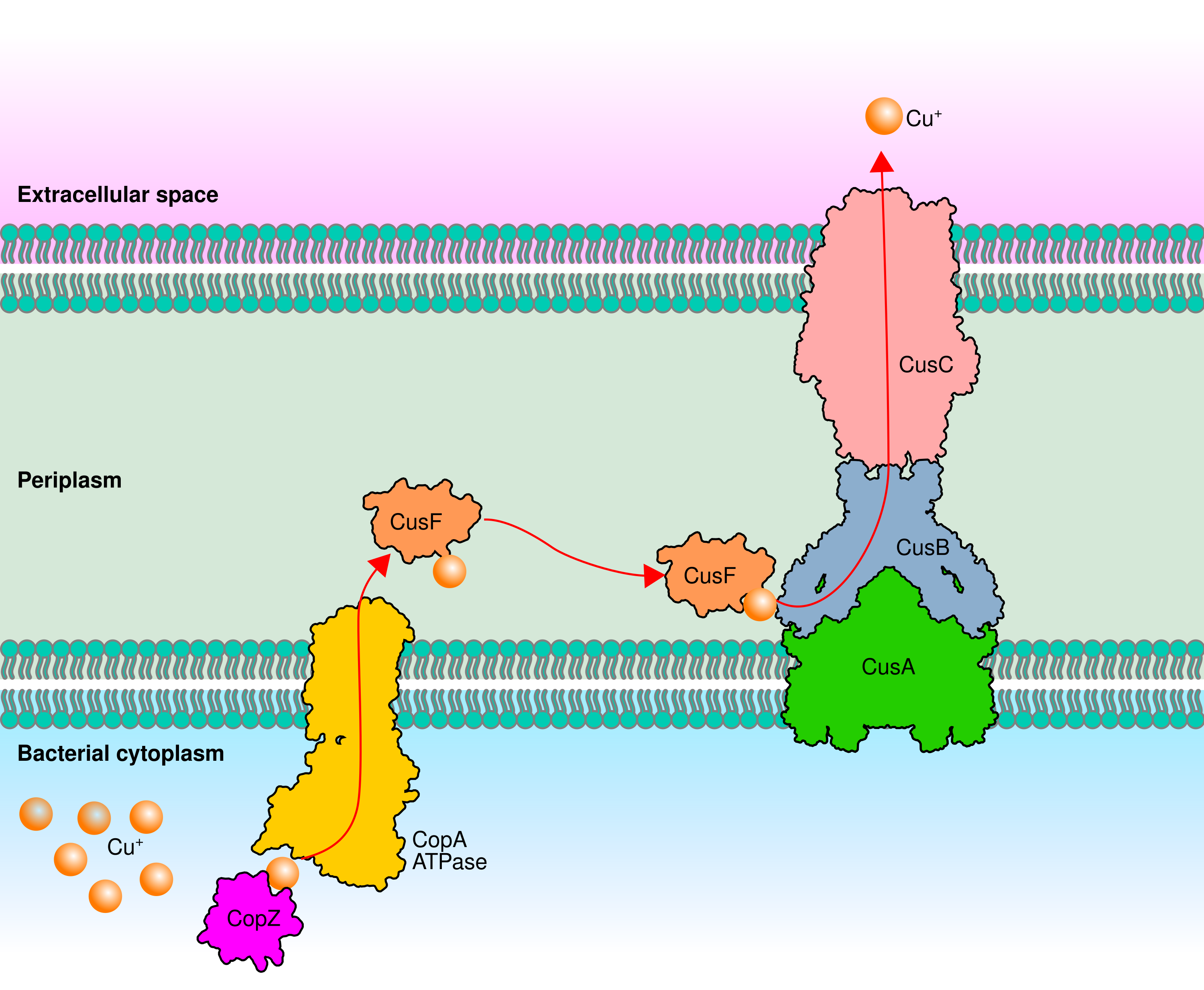

Supplement: Supplemental Information 2 — CopZ is a soluble cytoplasmic chaperone protein that binds and delivers Cu+ to the trans-membrane Cu-ATPase CopA. Subsequently, Cu+ is translocated from CopA to CusF, a soluble periplasmic chaperone protein that delivers Cu+ to the CusABC export system, which exports Cu+ to the extracellular space. [file peerj-11-14925-s002.png]
