# Supplementary material for: Five copper homeostasis gene clusters encode the Cu-efflux resistome of the highly copper-tolerant Methylorubrum extorquens AM1
Source: PeerJ. 2023 Feb 20;11:e14925. doi: 10.7717/peerj.14925 (PMC9948745; doi:10.7717/peerj.14925)
Supplement: Supplemental Information 3 — Strains: (a) PAFD, Rhizobium tropici CIAT899 (Rtr), R. etli CFN42 (Ret), R. freirei PRF81 (Rfr); and (b) PPFM, Methylorubrum extorquens AM1 (MeAM), Methylobacterium sp (Msp), M. radiotolerans JCM2831, M. nodulans ORS260 (Mno). [file peerj-11-14925-s003.pdf]

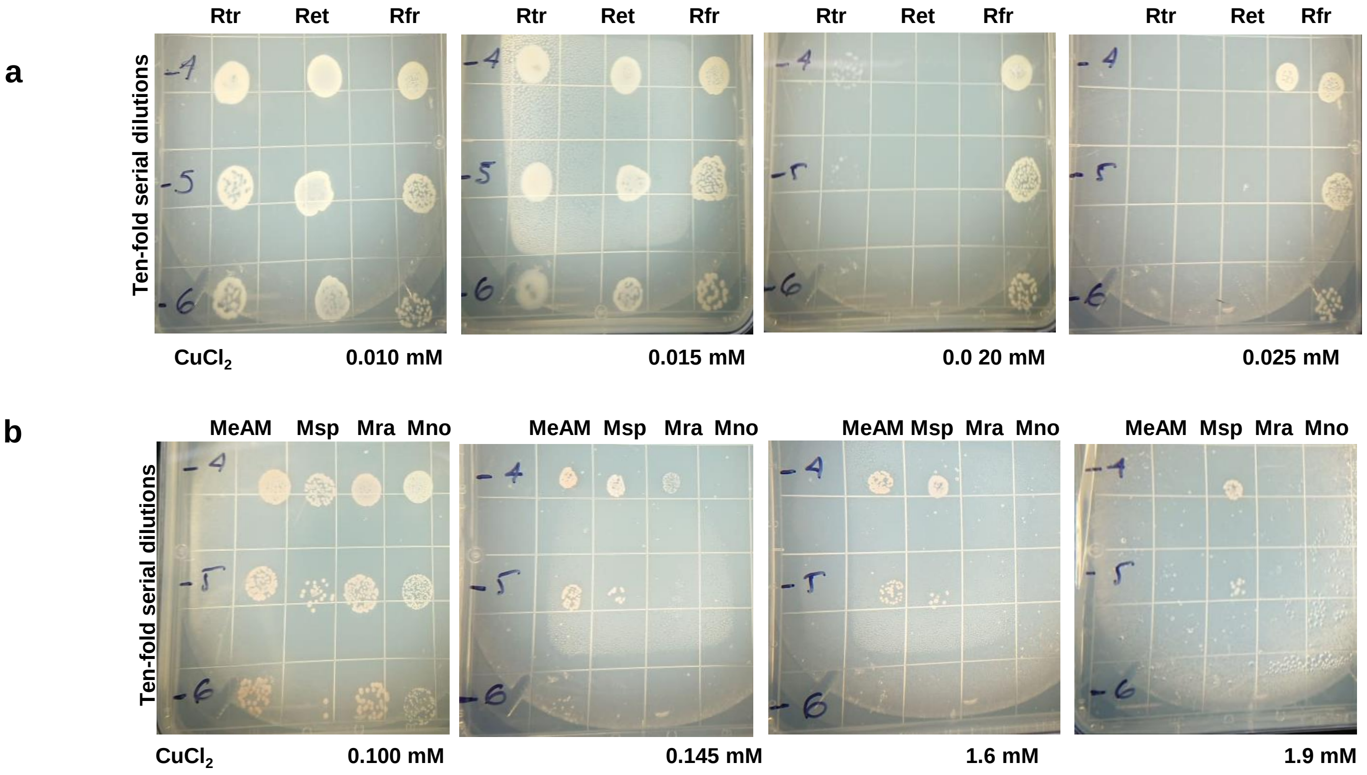

Fig. S1. Example of the dilution method used to estimate MICs of $CuCl_2$. Strains: (a) PAFD, *Rhizobium tropici* CIAT899 (Rtr), *R. etli* CFN42 (Ret), *R. freirei* PRF81 (Rfr); and (b) PPFM, *Methylorubrum extorquens* AM1 (MeAM), *Methylobacterium* sp (Msp), *M. radiotolerans* JCM2831, *M. nodulans* ORS260 (Mno).
