# Supplementary material for: Five copper homeostasis gene clusters encode the Cu-efflux resistome of the highly copper-tolerant Methylorubrum extorquens AM1
Source: PeerJ. 2023 Feb 20;11:e14925. doi: 10.7717/peerj.14925 (PMC9948745; doi:10.7717/peerj.14925)
Supplement: Supplemental Information 4 — Workflow for assessing MICs. (1) Bacterial cultures adjusted to OD620 = 0.3. (2) Tenfold serially diluted cultures. (3) 20 µl of each dilution were spotted on chemical defined medium with increasing concentrations of CuCl2. (4) Representative examples of minimal inhibitory concentrations of CuCl2 (in red color) that consistently prevented visible growth (arrow). Strains: M. sp (Methylobacterium sp), MeAM1 (Methylorubrum extorquens AM1), Rtr (Rhizobium tropici CIAT899), Ret (R. etli CFN42), Rfr (R. freirei). [file peerj-11-14925-s004.pdf]

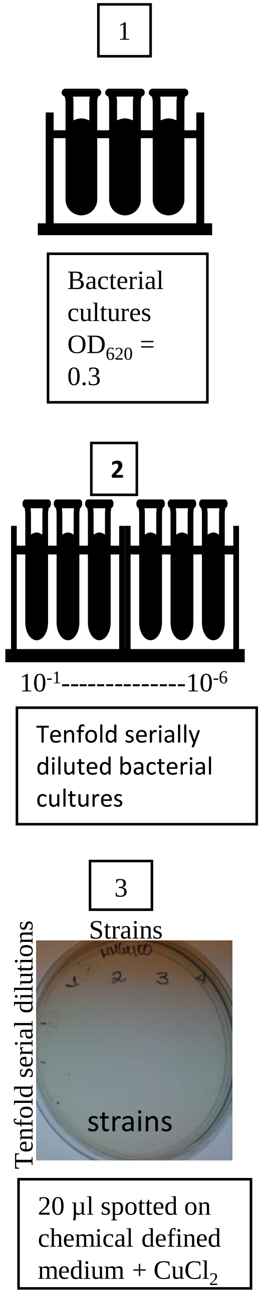

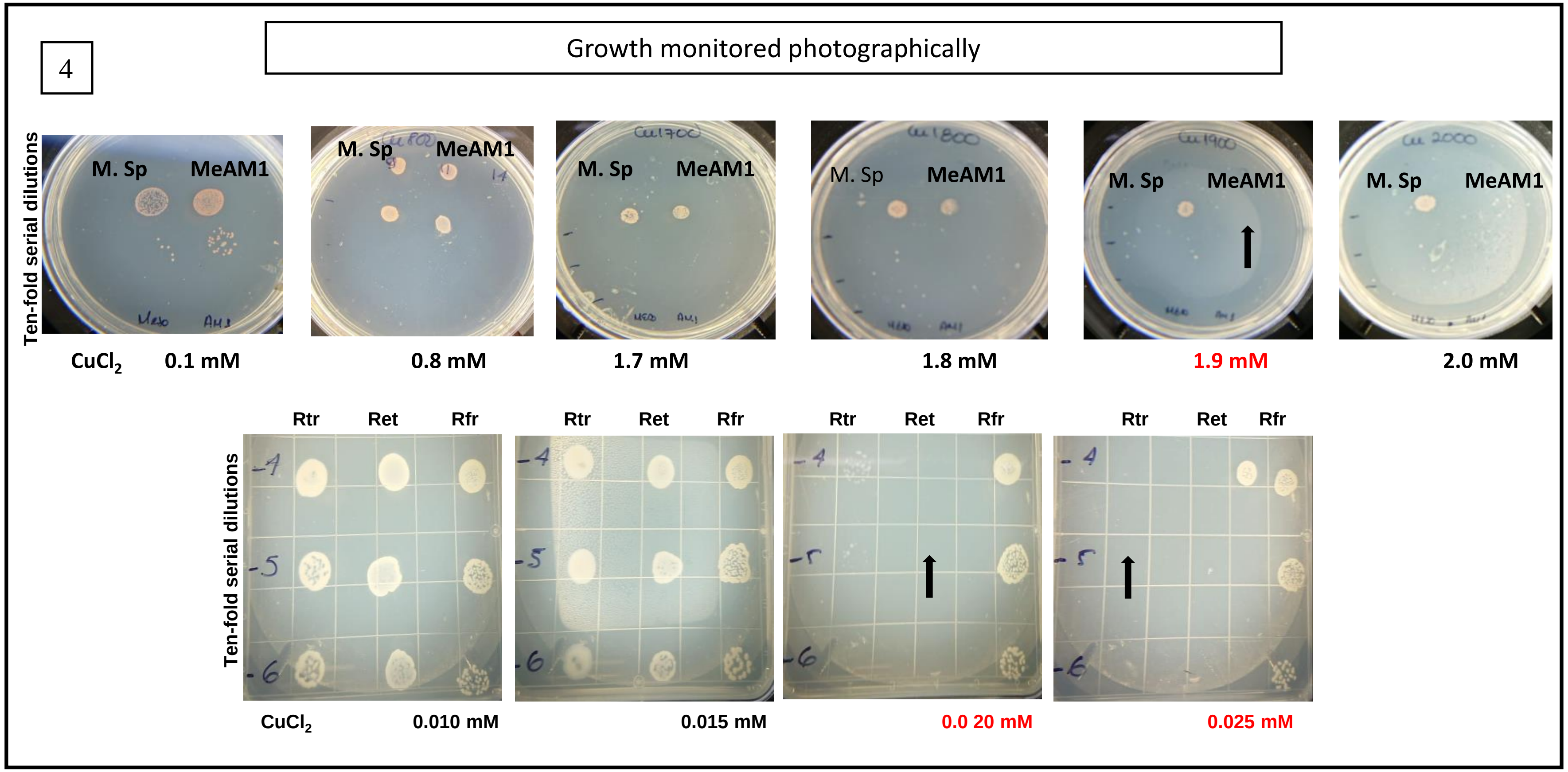

Fig. S2. Workflow for assessing MICs. (1) Bacterial cultures adjusted to OD_{620}= 0.3. (2) Tenfold serially diluted cultures. (3) 20 µl of each dilution were spotted on chemical defined medium with increasing concentrations of $CuCl_2$. (4) Representative examples of minimal inhibitory concentrations of $CuCl_2$ (in red color) that consistently prevented visible growth (arrow). Strains: M. sp (*Methylobacterium sp*), MeAM1 (*Methylorubrum extorquens AM1*), Rtr (*Rhizobium tropici* CIAT899), Ret (*R. etli* CFN42), Rfr (*R. freirei*).
