# Supplementary material for: Five copper homeostasis gene clusters encode the Cu-efflux resistome of the highly copper-tolerant Methylorubrum extorquens AM1
Source: PeerJ. 2023 Feb 20;11:e14925. doi: 10.7717/peerj.14925 (PMC9948745; doi:10.7717/peerj.14925)
Supplement: Supplemental Information 5 — The motifs involved in Cu binding and translocation shared among them are in bold. Full names of strains can be seen in Fig. 1. aThe multiple sequence alignment was performed with Clustal Omega version 1.2.4. at MBL-EBI (Clustal Omega < Multiple Sequence Alignment < EMBL-EBI). bThe TMH (black lines) were predicted with TOPCONS: Consensus prediction of membrane protein topology and signal peptides. [file peerj-11-14925-s005.pdf]

Fig.S2. Multiple amino acid sequences alignment[a] of transmembrane helices[b] (TMH)6, 7 and 8 of Cu-ATPases encoded in the genomes of PAFDs and PPFMs. The motifs involved in Cu binding and translocation shared among them are in bold. Full names of strains can be seen in Fig. 1

MloCh	IFAIVSAVSVLIIA**CPC**ALG	489
Rfr1Ch	AHALLAAVAVLIIA**CPC**ALG	485
RtrCh	AHALLAAVAVLIIA**CPC**ALG	483
Rle2pl11	ANGLLAAVAVLIIA**CPC**ALG	491
Retpe	ADALLAAVAVLIIA**CPC**ALG	490
Mno3p2	AHALVAAVAVLIIA**CPC**ALG	438
Rle1Ch	AHGLVAAVAVLIIA**CPC**ALG	389
SfGR1	AHGLVAAVAVLIIA**CPC**ALG	391
SfrNGR1Mpb	AHALVSAVAVLIIA**CPC**ALG	391
Sme1Mpa	THGLVAAVAVLIIA**CPC**ALG	355
MeAM2Ch	TFALLAAVAVLIIA**CPC**ALG	448
Mor2Ch	TFALLAAVAVLIIA**CPC**ALG	462
MeAM3Ch	TFALLAAVAVLIIA**CPC**ALG	455
MeAM5Mp	TFALLAAVAVLIIA**CPC**ALG	455
MeAM6Mp	TYALLAAVAVLIIA**CPC**ALG	459
Mno1Ch	TYGLLAAVAVLIIA**CPC**ALG	473
AcfeACH663	SIALVAAVAVLVVA**CPC**AMG	470
AcfeACH207	SVALVTAVAVLVVA**CPC**AMG	467
MeAM1Ch	GHALVAGISVLIIA**CPC**AMG	481
MexTK1Ch	GHALVAEISVLIIA**CPC**AMG	481
Mor1Ch	GPALVNAVAVLIIA**CPC**AMG	458
Mra1Ch	GPALVNAVAVLIIA**CPC**AMG	458
Rfr2Ch	TFALVNAVAVLIIA**CPC**AMG	476
Mno2p2	TFALVNAVAVLIIA**CPC**AMG	483
Rle3pl11	TFALVNAVAVLIIA**CPC**AMG	475
Sme2Mpa	TFALVNAVAVLIIA**CPC**AMG	475
Sme3Mpb	SFALVNAVAVLIIA**CPC**AMG	476
SfrNGR2Mpb	SFALVNAVAVLIIA**CPC**AMG	480
SfrNGR3Mpb	TFALVNGVAVLIIA**CPC**AMG	475
SfGR2	TFALVNSVAVLIIA**CPC**AMG	475
	**TMH6 CXC**	

MloCh	NLFFAFL**YNVLGVP**VAAGVLYPLTGMLLSPMLAAAA**MSLSS**VSVIANALRLRTLKL----	839
Rfr1Ch	NLAFAFG**YNALGVP**LAAGVLYPVFSLLLSPMIAAAA**MSLSS**VSVIANALRLRLAK-----	834
RtrCh	NLAFAFG**YNALGVP**LAAGVLYPIFGLLLSPMIAAAA**MSLSS**VSVIGNALRLRLAK-----	832
Rle2pl11	NLGFAFG**YNALGVP**VAAGMLYPIFGLLLSPMIAAAA**MSLSS**VSVISNALRLRFAKL----	841
Retpe	NLGFAFG**YNALGVP**VAAGVLYPIFGLLLSPMIAAAA**MSLSS**VSVIANALRLRFAKS----	840
Mno3p2	NLFFAFI**YNAAGVP**VAAGILYPSFGILLSPIIAAAA**MALSS**VSVIGNSLRLRSVRL----	788
Rle1Ch	NLFFAFI**YNAAGIP**VAAGVLYPAFGLLLSPIIAAAA**MALSS**VSVIGNSLRLRRSPLD---	740
SfGR1	NLFFAFI**YNAAGVP**VAAGVLYPAFGLLLSPIIAALA**MALSS**VSVIGNALRLRSAQI----	741
SfrNGR1Mpb	NLFFAFI**YNAAGVP**VAAGVLYPAFGLLLSPIIAAAA**MALSS**VSVIGNSLRLRTSQP----	741
Sme1Mpa	NLFFAFI**YNAAGVP**VAAGVLYPAFGLLLSPIIAAAA**MALSS**VSVIGNSLRLRSTRI----	705
MeAM2Ch	NLFFAFI**YNTAGVP**VAAGVLYPFLGILLSPVIAAAA**MALSS**VSVIGNALRLRGVGLDPSR	802
Mor2Ch	NLFFAFV**YNAAGVP**VAAGVLYPFLGILLSPIVAAAA**MALSS**VSVIGNALRLRATDLDPAP	816
MeAM3Ch	NLFFAFV**YNAAGVP**MAAGVLYPFLGILLSPVIAAAA**MALSS**VSVIGNALRLRATRLG---	806
MeAM5Mp	NLFFAFI**YNAAGVP**VAAGILYPFLGILLSPVIAAAA**MALSS**VSVIGNALRLRAARLG---	806
MeAM6Mp	NLFFAFI**YNAAGVP**VAAGVLFPFLGILLSPVIAAAA**MALSS**VSVIANALRLRAVRL----	809
Mno1Ch	NLFFAFI**YNAAGVP**VAAGVLYPVLGILLSPVIAAAA**MALSS**VSVIGNALRLRSLDI----	823
AcfeACH663	NLFWAFF**YNILLIP**VAAGVAVPI-GIHLNPMVAGVA**MGLSS**VFVLSNSLRLKRLKAYVPT	820
AcfeACH207	NLFWAFF**YNILLIP**IAAGVAAPI-GIHLNPMVAGVA**MGLSS**VFVLGNSLRLKRLKAYIPL	817
MeAM1Ch	NLFWAFA**YNAALIP**VAAGGLAVFGGPQLSPVLAAGA**MALSS**VFVVGNALRLKRAGGTA--	832
MexTK1Ch	NLFWAFA**YNAALIP**VAAGGLAVFGGPQLSPVLAAGA**MALSS**VFVVGNALRLKRAGGTA--	832
Mor1Ch	NLFWAFA**YNAALIP**VAAGVLVPFGGPALSPVLAAGA**MAFSS**VFVLGNALRLRRAGGRPAA	813
Mra1Ch	NLFWAFA**YNAALIP**VAAGVLVPFGGPALSPVLAAGA**MAFSS**VFVLGNALRLRRAGGRPAA	813

Rfr2Ch	NLFWAFA**YNTALVP**VAAGALYPTFDILLSPVFAAGA**MALSS**VFVLGNALRLRRFKLAD--	833
Mno2p2	NLFWAFA**YNTALIP**VAAGVLFPAFGILLSPVFAAGA**MALSS**VFVLGNALRLRRFKVAH--	835
Rle3pl11	NLFWAFV**YNASLVP**VAAGLLYPVNGTLLSPVFAAGA**MAMSS**VFVLGNALRLRRIEA----	824
Sme2Mpa	NLFWAFA**YNVSLVP**VAAGVLYPLNGTLLSPILAAAA**MAMSS**VFVLGNALRLRSVNPA---	826
Sme3Mpb	NLFWAFA**YNVSLIP**VAAGVLYPVTGILLSPIFAAAA**MAMSS**VFVLGNALRLKSVNPA---	827
SfrNGR2Mpb	NLFWAFA**YNTVLVP**VAAGALFPAYGLLLSPMIAAGA**MALSS**VFVLGNALRLKRFRAPMKF	834
SfrNGR3Mpb	NLFWAFA**YNVVLIP**VAAGALYPGYGMLLSPVFAAGA**MALSS**VFVVGNALRLKRFRGLERQ	829
SfGR2	NLFWAFA**YNVVLIP**VAAGALYPGYGVLLSPVFAAAA**MALSS**VFVVGNALRLKRFRGLERQ	829
	**YN(X_4)P** **TMH7** **M(X_3)S** **TMH8**	

[a]The multiple sequence alignment was performed with Clustal Omega version 1.2.4. at MBL-EBI (Clustal Omega < Multiple Sequence Alignment < EMBL-EBI)

[b]The TMH (black lines) were predicted with TOPCONS: Consensus prediction of membrane protein topology and signal peptides.
