# Supplementary material for: Five copper homeostasis gene clusters encode the Cu-efflux resistome of the highly copper-tolerant Methylorubrum extorquens AM1
Source: PeerJ. 2023 Feb 20;11:e14925. doi: 10.7717/peerj.14925 (PMC9948745; doi:10.7717/peerj.14925)
Supplement: Supplemental Information 6 — (a) NCBI Homepage resources toolbar menu. (1) Select protein in the resources box. (2) Paste the Refseq of CopA2 in the search box. (3) Click search. (b) In the protein page information click identical proteins tab. (c) Look up in the list the organism (red circle) and click the respective CDS region in nucleotides. (d) In the complete sequence page click graphics tab. (e) The graphics page shows the gene coding CopA2 protein. To get a broad view showing the copA2 neighborhood, adjust the display size in 50% with the zoom tool (red rectangle). Put the pointer over the Refseq to get information on the proteins encoded in each gene. To move upstream and downstream put the pointer over the displayed neighborhood, press left mouse button and drag. [file peerj-11-14925-s006.pdf]

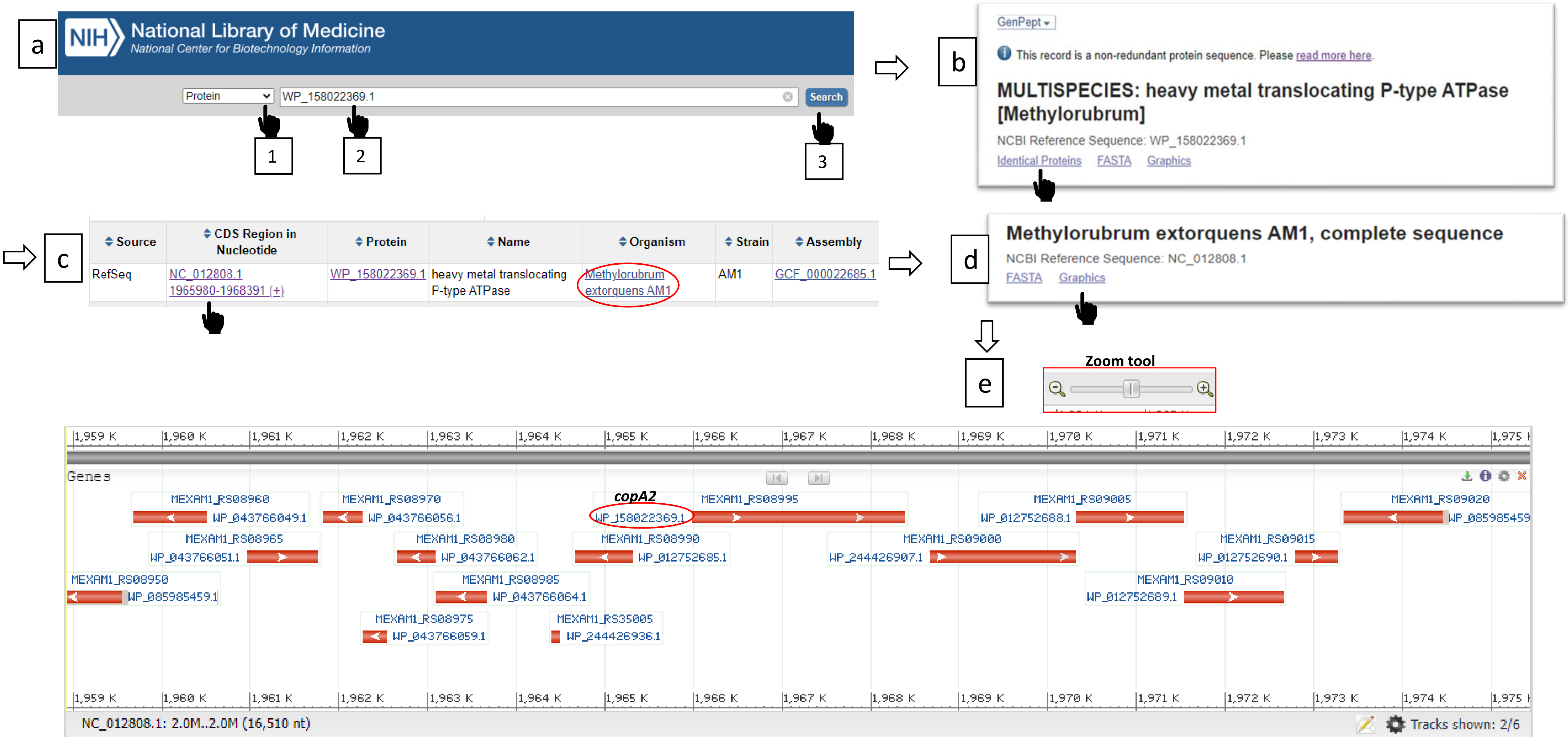

Fig. S3. Workflow for obtaining the gene neighborhood of *copA2* at NCBI (https://www.ncbi.nlm.nih.gov). a) NCBI Homepage resources toolbar menu. 1) Select protein in the resources box. 2) Paste the Refseq of CopA2 in the search box. 3) Click search. b) In the protein page information click identical proteins tab. c) Look up in the list the organism (red circle) and click the respective CDS region in nucleotides. d) In the complete sequence page click graphics tab. e) The graphics page shows the gene coding CopA2 protein. To get a broad view showing the *copA2* neighborhood , adjust the display size in 50% with the zoom tool (red rectangle). Put the pointer over the Refseq to get information on the proteins encoded in each gene. To move upstream and downstream put the pointer over the displayed neighborhood, press left mouse button and drag.
