# Supplementary material for: Five copper homeostasis gene clusters encode the Cu-efflux resistome of the highly copper-tolerant Methylorubrum extorquens AM1
Source: PeerJ. 2023 Feb 20;11:e14925. doi: 10.7717/peerj.14925 (PMC9948745; doi:10.7717/peerj.14925)
Supplement: Supplemental Information 12 [file peerj-11-14925-s012.docx]

Supplemental Table 5. Predicted functions encoded in the neighborhoods of *copA* genes from *M. extorquens* AM1.

| ***copA3* (Fig. 3a)** | | | |
| --- | --- | --- | --- |
| **Upstream neighboring gene product** | **Accession** | **NCBI Conserved domains** | **Accession** |
| UvrD-helicase domain-containing prote | [**WP_012752931.1**](https://www.ncbi.nlm.nih.gov/protein/WP_012752931.1) | ATP-dependent exoDNAse (exonuclease V) | [**cl40735**](https://www.ncbi.nlm.nih.gov/Structure/cdd/cddsrv.cgi?ascbin=8&maxaln=10&seltype=2&uid=cl40735) |
| PD-(D/E)XK nuclease family protein | [**WP_012752932.1**](https://www.ncbi.nlm.nih.gov/protein/WP_012752931.1) | CRISPR/Cas system-associated protein Cas4 | [**cl00641**](https://www.ncbi.nlm.nih.gov/Structure/cdd/cddsrv.cgi?ascbin=8&maxaln=10&seltype=2&uid=cl00641) |
| DEAD/DEAH box helicase | [**WP_012752933.1**](https://www.ncbi.nlm.nih.gov/protein/WP_012752933.1) | N-terminal helicase domain | [**cl28899**](https://www.ncbi.nlm.nih.gov/Structure/cdd/cddsrv.cgi?ascbin=8&maxaln=10&seltype=2&uid=cl28899) |
| ATP-binding protein | [**WP_012752934.1**](https://www.ncbi.nlm.nih.gov/protein/WP_012752934.1) | P-loop Domain of unknown function. Probably ATP-binding protein | [**pfam10923**](https://www.ncbi.nlm.nih.gov/Structure/cdd/cddsrv.cgi?ascbin=8&maxaln=10&seltype=2&uid=pfam10923) |
| Ter-dependent DNA processing | [**WP_012752935.1**](https://www.ncbi.nlm.nih.gov/protein/WP_012752935.1) | TerB_N and TerB_C  Stress response or phage defense | [**pfam13208**](https://www.ncbi.nlm.nih.gov/Structure/cdd/cddsrv.cgi?ascbin=8&maxaln=10&seltype=2&uid=pfam13208)  [**pfam15615**](https://www.ncbi.nlm.nih.gov/Structure/cdd/cddsrv.cgi?ascbin=8&maxaln=10&seltype=2&uid=pfam15615) |
| dNTP triphosphohydrolase | [**WP_012752937.1**](https://www.ncbi.nlm.nih.gov/protein/WP_012752937.1) | Triphosphohydrolase-like protein | [**cl35137**](https://www.ncbi.nlm.nih.gov/Structure/cdd/cddsrv.cgi?ascbin=8&maxaln=10&seltype=2&uid=cl35137) |
| Hypothetical protein | [**WP_009861980.1**](https://www.ncbi.nlm.nih.gov/protein/WP_009861980.1) | Hypothetical protein |  |
| Cytochrome C biogenesis protein CcdA | [**WP_012752938.1**](https://www.ncbi.nlm.nih.gov/protein/WP_012752938.1) | Cytochrome C biogenesis protein transmembrane region | [**PF02683**](https://pfam.xfam.org/family/PF02683) |
| Murein L,D-transpeptidase | [**WP_003598662.1**](https://www.ncbi.nlm.nih.gov/protein/WP_003598662.1) | Murein synthesis | [**COG3034**](https://www.ncbi.nlm.nih.gov/Structure/cdd/cddsrv.cgi?ascbin=8&maxaln=10&seltype=2&uid=COG3034) |
| Signal peptidase II | [**WP_009864915.1**](https://www.ncbi.nlm.nih.gov/protein/WP_009864915.1) | Signal peptidase II | [**pfam01252**](https://www.ncbi.nlm.nih.gov/Structure/cdd/cddsrv.cgi?ascbin=8&maxaln=10&seltype=2&uid=pfam01252) |
| cadmium-translocating P-type ATPase | [**WP_012752941.1**](https://www.ncbi.nlm.nih.gov/protein/WP_012752941.1) | Heavy-metal-associated domain | [**cd00371**](https://www.ncbi.nlm.nih.gov/Structure/cdd/cddsrv.cgi?ascbin=8&maxaln=10&seltype=2&uid=cd00371) |
| Helix-turn-helix domain-containing protein | [**WP_012752942.1**](https://www.ncbi.nlm.nih.gov/protein/WP_012752942.1) | Transcription regulator MerR, CadR, PbrR | [**cd04785**](https://www.ncbi.nlm.nih.gov/Structure/cdd/cddsrv.cgi?ascbin=8&maxaln=10&seltype=2&uid=cd04785) |
| **CopA3 heavy metal translocating P-type ATPase** | [**WP_238231531.1**](https://www.ncbi.nlm.nih.gov/protein/WP_238231531.1?report=graph)  **Formely:**  [**WP_012752944.1**](https://www.ncbi.nlm.nih.gov/protein/WP_012752944.1?report=genpept) | P-type_ATPase_Cu-like | [**cd02094**](https://www.ncbi.nlm.nih.gov/Structure/cdd/cddsrv.cgi?ascbin=8&maxaln=10&seltype=2&uid=cd02094) |
| **Downstream neighboring gene product** | **Accession** | **NCBI Conserved domains** | **Accession** |
| Domain of unknown function | [**WP_012752945.1**](https://www.ncbi.nlm.nih.gov/protein/WP_012752945.1) | DUF305 | [**cl21583**](https://www.ncbi.nlm.nih.gov/Structure/cdd/cddsrv.cgi?ascbin=8&maxaln=10&seltype=2&uid=cl21583) |
| Four-helix bundle copper-binding protein | [**WP_009865580.1**](https://www.ncbi.nlm.nih.gov/protein/WP_009865580.1) | Cysteine-rich 4 helical bundle widely conserved in bacteria | [**cd08026**](https://www.ncbi.nlm.nih.gov/Structure/cdd/cddsrv.cgi?ascbin=8&maxaln=10&seltype=2&uid=cd08026) |
| Domain of unknown function | [**WP_009865581.1**](https://www.ncbi.nlm.nih.gov/protein/WP_009865581.1) | DUF305 | [**cl21583**](https://www.ncbi.nlm.nih.gov/Structure/cdd/cddsrv.cgi?ascbin=8&maxaln=10&seltype=2&uid=cl21583) |
| Hypothetical protein | [**WP_012752946.1**](https://www.ncbi.nlm.nih.gov/protein/WP_012752946.1) | No conserved domains |  |
| Efflux RND transporter permease subunit | [**WP_012752947.1**](https://www.ncbi.nlm.nih.gov/protein/WP_012752947.1) | Cu/Ag efflux pump CusA | [**cl34658**](https://www.ncbi.nlm.nih.gov/Structure/cdd/cddsrv.cgi?ascbin=8&maxaln=10&seltype=2&uid=cl34658) |
| Efflux RND transporter periplasmic adaptor subunit | [**WP_012455885.1**](https://www.ncbi.nlm.nih.gov/protein/WP_012455885.1) | Membrane Fusion Protein | [**cl36796**](https://www.ncbi.nlm.nih.gov/Structure/cdd/cddsrv.cgi?ascbin=8&maxaln=10&seltype=2&uid=cl36796) |
| Hypothetical protein | [**WP_012752950.1**](https://www.ncbi.nlm.nih.gov/protein/WP_012752950.1) | No conserved domains |  |
| Copper chaperone | [**WP_003598673.1**](https://www.ncbi.nlm.nih.gov/protein/WP_003598673.1) | CopZ | [**COG2608**](https://www.ncbi.nlm.nih.gov/Structure/cdd/cddsrv.cgi?ascbin=8&maxaln=10&seltype=2&uid=COG2608) |
| Hypothetical protein | [**WP_009865907.1**](https://www.ncbi.nlm.nih.gov/protein/WP_009865907.1) | No conserved domains |  |
| Nickel/cobalt efflux transporter RcnA | [**WP_012752951.1**](https://www.ncbi.nlm.nih.gov/protein/WP_012752951.1) | Family of integral membrane proteins | [**cl21514**](https://www.ncbi.nlm.nih.gov/Structure/cdd/cddsrv.cgi?ascbin=8&maxaln=10&seltype=2&uid=cl21514) |
| Transcriptional regulators RcnR | [**WP_009866592.1**](https://www.ncbi.nlm.nih.gov/protein/WP_009866592.1) | RcnR-FrmR-like_DUF156 | [**cd10153**](https://www.ncbi.nlm.nih.gov/Structure/cdd/cddsrv.cgi?ascbin=8&maxaln=10&seltype=2&uid=cd10153) |
| DUF1289 domain-containing protein | [**WP_012752952.1**](https://www.ncbi.nlm.nih.gov/protein/WP_012752952.1) | DUF1289 | [**pfam06945**](https://www.ncbi.nlm.nih.gov/Structure/cdd/cddsrv.cgi?ascbin=8&maxaln=10&seltype=2&uid=pfam06945) |
| Type II restriction endonuclease | [**GCF_000022685.1-MEXAM1_RS12375**](https://mistdb.com/genes/GCF_000022685.1-MEXAM1_RS12375) | No conserved domains |  |
| MerR family DNA-binding protein | [**WP_009866299.1**](https://www.ncbi.nlm.nih.gov/protein/WP_009866299.1) | Helix-Turn-Helix DNA binding domain, MerR family | [**cl02600**](https://www.ncbi.nlm.nih.gov/Structure/cdd/cddsrv.cgi?ascbin=8&maxaln=10&seltype=2&uid=cl02600) |
| Heavy-metal-associated domain-containing protein | [**WP_043766175.1**](https://www.ncbi.nlm.nih.gov/protein/WP_043766175.1) | Heavy-metal-associated domain (HMA) | [**cd00371**](https://www.ncbi.nlm.nih.gov/Structure/cdd/cddsrv.cgi?ascbin=8&maxaln=10&seltype=2&uid=cd00371) |
| Cation-translocating P-type ATPase | [**WP_012752954.1**](https://www.ncbi.nlm.nih.gov/protein/WP_012752954.1) | ZntA | [**COG2217**](https://www.ncbi.nlm.nih.gov/Structure/cdd/cddsrv.cgi?ascbin=8&maxaln=10&seltype=2&uid=COG2217) |
| Heavy-metal-associated domain-containing protein | [**WP_012752955.1**](https://www.ncbi.nlm.nih.gov/protein/WP_012752955.1) | Heavy-metal-associated domain (HMA) | [**cd00371**](https://www.ncbi.nlm.nih.gov/Structure/cdd/cddsrv.cgi?ascbin=8&maxaln=10&seltype=2&uid=cd00371) |
| Thiol:disulfide interchange protein | [**WP_009866213.1**](https://www.ncbi.nlm.nih.gov/protein/WP_009866213.1) | Sulfite exporter TauE/SafE | [**cl21514**](https://www.ncbi.nlm.nih.gov/Structure/cdd/cddsrv.cgi?ascbin=8&maxaln=10&seltype=2&uid=cl21514) |
| Heavy-metal-associated domain-containing protein | [**WP_012752956.1**](https://www.ncbi.nlm.nih.gov/protein/WP_012752956.1) | Copper chaperone CopZ | [**COG2608**](https://www.ncbi.nlm.nih.gov/Structure/cdd/cddsrv.cgi?ascbin=8&maxaln=10&seltype=2&uid=COG2608) |
| MFS Transporter | [**WP_012752957.1**](https://www.ncbi.nlm.nih.gov/protein/WP_012752957.1) | MFS efflux pump | [**cd17324**](https://www.ncbi.nlm.nih.gov/Structure/cdd/cddsrv.cgi?ascbin=8&maxaln=10&seltype=2&uid=cd17324) |
|  |  |  |  |
| ***copA4* (Fig. 3b)** | | | |
| **Upstream neighboring gene product** | **Accession** | **NCBI Conserved domains** | **Accession** |
| Hypothetical protein | [**WP_003598644.1**](https://www.ncbi.nlm.nih.gov/protein/WP_003598644.1) | No conserved domains |  |
| Hypothetical protein | [**WP_003598646.1**](https://www.ncbi.nlm.nih.gov/protein/WP_003598646.1) | No conserved domains |  |
| Hypothetical protein | [**WP_012753608.1**](https://www.ncbi.nlm.nih.gov/protein/WP_012753608.1?report=genpept) | No conserved domains |  |
| IS110 family transposase | [**WP_049832675.1**](https://www.ncbi.nlm.nih.gov/protein/WP_049832675.1) | IS110 family transposase | [**NF033542**](https://www.ncbi.nlm.nih.gov/Structure/cdd/cddsrv.cgi?ascbin=8&maxaln=10&seltype=2&uid=NF033542) |
| IS3 family transposase | [**GCF_000021845.1-MCHL_RS05200**](https://mistdb.com/genes/GCF_000021845.1-MCHL_RS05200) |  |  |
| IS630 family transposase | [**WP_085985055.1**](https://www.ncbi.nlm.nih.gov/protein/WP_085985055.1) | IS630 family transposase | [**NF033545**](https://www.ncbi.nlm.nih.gov/Structure/cdd/cddsrv.cgi?ascbin=8&maxaln=10&seltype=2&uid=NF033545) |
| Transposase | [**GCF_000021845.1-MCHL_RS28720**](https://mistdb.com/genes/GCF_000021845.1-MCHL_RS28720) |  |  |
| Hypothetical protein | [**WP_012753609.1**](https://www.ncbi.nlm.nih.gov/protein/WP_012753609.1?report=genpept) | No conserved domains |  |
| Cytochrome C biogenesis protein | [**WP_080515519.1**](https://www.ncbi.nlm.nih.gov/protein/WP_080515519.1?report=genpept) | Cytochrome C biogenesis protein transmembrane region | [**PF02683**](https://pfam.xfam.org/family/PF02683) |
| Murein L,D-transpeptidase | [**WP_003598654.1**](https://www.ncbi.nlm.nih.gov/protein/WP_003598654.1) | Murein L,D-transpeptidase YafK | [**COG3034**](https://www.ncbi.nlm.nih.gov/Structure/cdd/cddsrv.cgi?ascbin=8&maxaln=10&seltype=2&uid=COG3034) |
| Signal peptidase II | [**ACS43110.1**](https://www.ncbi.nlm.nih.gov/protein/ACS43110.1) | Signal peptidase II | [**pfam01252**](https://www.ncbi.nlm.nih.gov/Structure/cdd/cddsrv.cgi?ascbin=8&maxaln=10&seltype=2&uid=pfam01252) |
| cadmium-translocating P-type ATPase | [**WP_003598656.1**](https://www.ncbi.nlm.nih.gov/protein/WP_003598656.1) | Conserved domain P-type_ATPase_Pb_Zn_Cd2-like | [**cd00371**](https://www.ncbi.nlm.nih.gov/Structure/cdd/cddsrv.cgi?ascbin=8&maxaln=10&seltype=2&uid=cd00371) |
| Helix-turn-helix domain-containing protein | [**WP_003598658.1**](https://www.ncbi.nlm.nih.gov/protein/WP_003598658.1) | HTH_CadR-PbrR-like | [**cd04785**](https://www.ncbi.nlm.nih.gov/Structure/cdd/cddsrv.cgi?ascbin=8&maxaln=10&seltype=2&uid=cd04785) |
| Hypothetical protein | [**WP_012753611.1**](https://www.ncbi.nlm.nih.gov/protein/WP_012753611.1?report=genpept) | No conserved domains |  |
| **CopA4 Cation-transporting P-type ATPase** | [**ACS43114.1**](https://www.ncbi.nlm.nih.gov/protein/ACS43114.1)  **Formely:**  [**WP_003598660.1**](https://www.ncbi.nlm.nih.gov/protein/WP_003598660.1?report=genpept) | P-type_ATPase_Cu-like | [**cd02094**](https://www.ncbi.nlm.nih.gov/Structure/cdd/cddsrv.cgi?ascbin=8&maxaln=10&seltype=2&uid=cd02094) |
| **Downstream neighboring gene product** | **Accession** | **NCBI Conserved domains** | **Accession** |
| DUF305 domain-containing protein | [**WP_003598662.1**](https://www.ncbi.nlm.nih.gov/protein/WP_003598662.1) | Domain of unknown function (DUF305) | [**COG3544**](https://www.ncbi.nlm.nih.gov/Structure/cdd/cddsrv.cgi?ascbin=8&maxaln=10&seltype=2&uid=COG3544&querygi=489694493&aln=2,54,91,12,67,103,87) |
| four-helix bundle copper-binding protein | [**WP_003598663.1**](https://www.ncbi.nlm.nih.gov/protein/WP_003598663.1) | Cysteine-rich 4 helical bundle | [**cd08026**](https://www.ncbi.nlm.nih.gov/Structure/cdd/cddsrv.cgi?ascbin=8&maxaln=10&seltype=2&uid=cd08026) |
| DUF305 domain-containing protein | [**WP_003598665.1**](https://www.ncbi.nlm.nih.gov/protein/WP_003598665.1) | Domain of unknown function (DUF305) | [**COG3544**](https://www.ncbi.nlm.nih.gov/Structure/cdd/cddsrv.cgi?ascbin=8&maxaln=10&seltype=2&uid=COG3544&querygi=489694493&aln=2,54,91,12,67,103,87) |
| Hypothetical protein | [**WP_003598666.1**](https://www.ncbi.nlm.nih.gov/protein/WP_003598666.1) | No conserved domains |  |
| Efflux RND transporter permease subunit | [**WP_003598668.1**](https://www.ncbi.nlm.nih.gov/protein/WP_003598668.1) | Cu/Ag efflux pump CusA | [**cl34658**](https://www.ncbi.nlm.nih.gov/Structure/cdd/cddsrv.cgi?ascbin=8&maxaln=10&seltype=2&uid=cl34658) |
| Efflux RND transporter periplasmic adaptor subunit | [**WP_003598670.1**](https://www.ncbi.nlm.nih.gov/protein/WP_003598670.1) | Membrane Fusion Protein cluster 2 (function with RND porters) | [**cl36796**](https://www.ncbi.nlm.nih.gov/Structure/cdd/cddsrv.cgi?ascbin=8&maxaln=10&seltype=2&uid=cl36796) |
| Hypothetical protein | [**WP_012606013.1**](https://www.ncbi.nlm.nih.gov/protein/WP_012606013.1) | No conserved domains |  |
| Heavy-metal-associated domain-containing protein | [**WP_003598673.1**](https://www.ncbi.nlm.nih.gov/protein/WP_003598673.1) | Copper chaperone CopZ | [**COG2608**](https://www.ncbi.nlm.nih.gov/Structure/cdd/cddsrv.cgi?ascbin=8&maxaln=10&seltype=2&uid=COG2608) |
| NYN domain-containing protein | [**WP_003598674.1**](https://www.ncbi.nlm.nih.gov/protein/WP_003598674.1) | PIN_NicB-like | [**cd18722**](https://www.ncbi.nlm.nih.gov/Structure/cdd/cddsrv.cgi?ascbin=8&maxaln=10&seltype=2&uid=cd18722) |
| Metal-binding protein | [**WP_003598675.1**](https://www.ncbi.nlm.nih.gov/protein/WP_003598675.1) | DUF411 super family | [**cl23841**](https://www.ncbi.nlm.nih.gov/Structure/cdd/cddsrv.cgi?ascbin=8&maxaln=10&seltype=2&uid=cl23841) |
| CusA/CzcA family heavy metal efflux RND transporte | [**WP_003598676.1**](https://www.ncbi.nlm.nih.gov/protein/WP_003598676.1) | Cu/Ag efflux pump CusA | [**cl34658**](https://www.ncbi.nlm.nih.gov/Structure/cdd/cddsrv.cgi?ascbin=8&maxaln=10&seltype=2&uid=cl34658) |
| Efflux transporter periplasmic adaptor subunit | [**GCF_000021845.1-MCHL_RS05315**](https://mistdb.com/genes/GCF_000021845.1-MCHL_RS05315) |  |  |
| IS3 family transposase | [**WP_085985478.1**](https://www.ncbi.nlm.nih.gov/protein/WP_085985478.1) | transpos_IS3 | [**NF033516**](https://www.ncbi.nlm.nih.gov/Structure/cdd/cddsrv.cgi?ascbin=8&maxaln=10&seltype=2&uid=NF033516) |
|  |  |  |  |
| ***copA5* (Fig. 4a)** | | | |
| **Upstream neighboring gene product** | **Accession** | **NCBI Conserved domains** | **Accession** |
| Hypothetical protein | [**WP_012753567.1**](https://www.ncbi.nlm.nih.gov/protein/WP_012753567.1) | No conserved domains |  |
| Hypothetical protein | [**WP_012753568.1**](https://www.ncbi.nlm.nih.gov/protein/WP_012753568.1) | No conserved domains |  |
| Hypothetical protein | [**WP_012753569.1**](https://www.ncbi.nlm.nih.gov/protein/WP_012753569.1) | No conserved domains |  |
| Hypothetical protein | [**WP_012753570.1**](https://www.ncbi.nlm.nih.gov/protein/WP_012753570.1?report=genpept) | No conserved domains |  |
| Hypothetical protein | [**WP_043767476.1**](https://www.ncbi.nlm.nih.gov/protein/WP_043767476.1?report=genpept) | No conserved domains |  |
| HRDC domain-containing protein | [**WP_012753572.1**](https://www.ncbi.nlm.nih.gov/protein/WP_012753572.1?report=genpept) | Superfamily II DNA helicase RecQ | [**cl33925**](https://www.ncbi.nlm.nih.gov/Structure/cdd/cddsrv.cgi?ascbin=8&maxaln=10&seltype=2&uid=cl33925) |
| Hypothetical protein | [**WP_012605960.1**](https://www.ncbi.nlm.nih.gov/protein/WP_012605960.1) | No conserved domains |  |
| CBS domain-containing protein | [**WP_080515517.1**](https://www.ncbi.nlm.nih.gov/protein/WP_080515517.1) | Cystathionine beta-synthase (CBS pair) domains superfamily | [**cl15354**](https://www.ncbi.nlm.nih.gov/Structure/cdd/cddsrv.cgi?ascbin=8&maxaln=10&seltype=2&uid=cl15354) |
| Hypothetical protein | [**WP_012753574.1**](https://www.ncbi.nlm.nih.gov/protein/WP_012753574.1?report=genpept) | No conserved domains |  |
| Efflux RND transporter periplasmic adaptor subunit | [**WP_003600345.1**](https://www.ncbi.nlm.nih.gov/protein/WP_003600345.1) | Membrane Fusion Protein cluster 2 (function with RND porters) | [**cl36796**](https://www.ncbi.nlm.nih.gov/Structure/cdd/cddsrv.cgi?ascbin=8&maxaln=10&seltype=2&uid=cl36796) |
| Efflux RND transporter permease subunit | [**WP_012753575.1**](https://www.ncbi.nlm.nih.gov/protein/WP_012753575.1) | Cu/Ag efflux pump CusA | [**cl34658**](https://www.ncbi.nlm.nih.gov/Structure/cdd/cddsrv.cgi?ascbin=8&maxaln=10&seltype=2&uid=cl34658) |
| Hypothetical protein | [**WP_003600349.1**](https://www.ncbi.nlm.nih.gov/protein/WP_003600349.1) | No conserved domains |  |
| DUF305 domain-containing protein | [**WP_012753576.1**](https://www.ncbi.nlm.nih.gov/protein/WP_012753576.1) | DUF305 domain-containing protein | [**COG3544**](https://www.ncbi.nlm.nih.gov/Structure/cdd/cddsrv.cgi?ascbin=8&maxaln=10&seltype=2&uid=COG3544&querygi=489694493&aln=2,54,91,12,67,103,87) |
| Four-helix bundle copper-binding protein | [**WP_003600351.1**](https://www.ncbi.nlm.nih.gov/protein/WP_003600351.1) | Cysteine-rich 4 helical bundle | [**cd08026**](https://www.ncbi.nlm.nih.gov/Structure/cdd/cddsrv.cgi?ascbin=8&maxaln=10&seltype=2&uid=cd08026) |
| Hypothetical protein | [**WP_003600352.1**](https://www.ncbi.nlm.nih.gov/protein/WP_003600352.1) | No conserved domains |  |
| **CopA5** **putative cation transporting P-type ATPase** | [**ACS43019.1**](https://www.ncbi.nlm.nih.gov/protein/ACS43019.1)  **Formely:**  [**WP_012753577.1**](https://www.ncbi.nlm.nih.gov/protein/WP_012753577.1?report=genpept) | P-type_ATPase_Cu-like | [**cd02094**](https://www.ncbi.nlm.nih.gov/Structure/cdd/cddsrv.cgi?ascbin=8&maxaln=10&seltype=2&uid=cd02094) |
| **Downstream neighboring gene product** | **Accession** | **NCBI Conserved domains** | **Accession** |
| Metal-binding protein | [**WP_012605964.1**](https://www.ncbi.nlm.nih.gov/protein/WP_012605964.1) | Protein of unknown function, DUF411 | [**cl23841**](https://www.ncbi.nlm.nih.gov/Structure/cdd/cddsrv.cgi?ascbin=8&maxaln=10&seltype=2&uid=cl23841) |
| Hypothetical protein | [**WP_015821500.1**](https://www.ncbi.nlm.nih.gov/protein/WP_015821500.1) | No conserved domains |  |
| Hypothetical protein | [**WP_012753578.1**](https://www.ncbi.nlm.nih.gov/protein/WP_012753578.1?report=genpept) | No conserved domains |  |
| Hypothetical protein | [**WP_012753579.1**](https://www.ncbi.nlm.nih.gov/protein/WP_012753579.1?report=genpept) | No conserved domains |  |
| Hypothetical protein | [**WP_003600358.1**](https://www.ncbi.nlm.nih.gov/protein/WP_003600358.1) | No conserved domains |  |
| Hypothetical protein | [**WP_012753581.1**](https://www.ncbi.nlm.nih.gov/protein/WP_012753581.1?report=genpept) | No conserved domains |  |
| Hypothetical protein | [**WP_009864177.1**](https://www.ncbi.nlm.nih.gov/protein/WP_009864177.1?report=genpept) | No conserved domains |  |
| TonB-dependent receptor | [**WP_043767482.1**](https://www.ncbi.nlm.nih.gov/protein/WP_043767482.1) | OM_channels super family | [**cl21487**](https://www.ncbi.nlm.nih.gov/Structure/cdd/cddsrv.cgi?ascbin=8&maxaln=10&seltype=2&uid=cl21487) |
| Hypothetical protein | [**WP_003600362.1**](https://www.ncbi.nlm.nih.gov/protein/WP_003600362.1?report=genpept) | No conserved domains |  |
| Hypothetical protein | [**WP_003600363.1**](https://www.ncbi.nlm.nih.gov/protein/WP_003600363.1?report=genpept) | No conserved domains |  |
| Metal-sensitive transcriptional regulator | [**WP_012753584.1**](https://www.ncbi.nlm.nih.gov/protein/WP_012753584.1?report=genpept) | CsoR-like_DUF156 | [**cd10148**](https://www.ncbi.nlm.nih.gov/Structure/cdd/cddsrv.cgi?ascbin=8&maxaln=10&seltype=2&uid=cd10148) |
| Heavy-metal-associated domain-containing protein | [**WP_082222840.1**](https://www.ncbi.nlm.nih.gov/protein/WP_082222840.1?report=genpept) | CopZ | [**COG2608**](https://www.ncbi.nlm.nih.gov/Structure/cdd/cddsrv.cgi?ascbin=8&maxaln=10&seltype=2&uid=COG2608) |
| Hypothetical protein | [**WP_003600369.1**](https://www.ncbi.nlm.nih.gov/protein/WP_003600369.1) | No conserved domains |  |
| Calcium-binding protein | [**WP_012605967.1**](https://www.ncbi.nlm.nih.gov/protein/WP_012605967.1) | RTX_toxin super family | [**cl41537**](https://www.ncbi.nlm.nih.gov/Structure/cdd/cddsrv.cgi?ascbin=8&maxaln=10&seltype=2&uid=cl41537) |
|  |  |  |  |
| ***CopA1* (Fig. 4b)** | | | |
| **Upstream neighboring gene product** | **Accession** | **NCBI Conserved domains** | **Accession** |
| Dihydroxy-acid dehydratase | [**WP_003597797.1**](https://www.ncbi.nlm.nih.gov/protein/WP_003597797.1) | dihydroxy-acid dehydratase | [**PRK12448**](https://www.ncbi.nlm.nih.gov/Structure/cdd/cddsrv.cgi?ascbin=8&maxaln=10&seltype=2&uid=PRK12448) |
| DUF1611 domain-containing protein | [**WP_003597798.1**](https://www.ncbi.nlm.nih.gov/protein/WP_003597798.1) | Uncharacterized conserved protein, NAD-dependent epimerase/dehydratase family | [**cl34593**](https://www.ncbi.nlm.nih.gov/Structure/cdd/cddsrv.cgi?ascbin=8&maxaln=10&seltype=2&uid=cl34593) |
| Dipeptide epimerase | [**WP_003597800.1**](https://www.ncbi.nlm.nih.gov/protein/WP_003597800.1) | L-Ala-DL-Glu_epimerase | [**cd03319**](https://www.ncbi.nlm.nih.gov/Structure/cdd/cddsrv.cgi?ascbin=8&maxaln=10&seltype=2&uid=cd03319) |
| Transglycosylase SLT domain-containing protein | [**WP_012753100.1**](https://www.ncbi.nlm.nih.gov/protein/WP_012753100.1?report=genpept) | lysozyme-like domains | [**cl00222**](https://www.ncbi.nlm.nih.gov/Structure/cdd/cddsrv.cgi?ascbin=8&maxaln=10&seltype=2&uid=cl00222) |
| Inositol-3-phosphate synthase | [**WP_003597802.1**](https://www.ncbi.nlm.nih.gov/protein/WP_003597802.1) | Myo-inositol-1-phosphate synthase family | [**pfam07994**](https://www.ncbi.nlm.nih.gov/Structure/cdd/cddsrv.cgi?ascbin=8&maxaln=10&seltype=2&uid=pfam07994) |
| Hypothetical protein | [**WP_012753101.1**](https://www.ncbi.nlm.nih.gov/protein/WP_012753101.1?report=genpept) | Metal dependent phosphohydrolases with conserved 'HD' motif | [**cl21469**](https://www.ncbi.nlm.nih.gov/Structure/cdd/cddsrv.cgi?ascbin=8&maxaln=10&seltype=2&uid=cl21469) |
| Methyltransferase domain-containing protein | [**WP_003597807.1**](https://www.ncbi.nlm.nih.gov/protein/WP_003597807.1) | S-adenosylmethionine-dependent methyltransferases | [**cl17173**](https://www.ncbi.nlm.nih.gov/Structure/cdd/cddsrv.cgi?ascbin=8&maxaln=10&seltype=2&uid=cl17173) |
| Gfo/Idh/MocA family oxidoreductase | [**WP_012753102.1**](https://www.ncbi.nlm.nih.gov/protein/WP_012753102.1) | Predicted dehydrogenase | [**COG0673**](https://www.ncbi.nlm.nih.gov/Structure/cdd/cddsrv.cgi?ascbin=8&maxaln=10&seltype=2&uid=COG0673) |
| YcaO-like family protein | [**WP_003597810.1**](https://www.ncbi.nlm.nih.gov/protein/WP_003597810.1) | YcaO cyclodehydratase | [**pfam02624**](https://www.ncbi.nlm.nih.gov/Structure/cdd/cddsrv.cgi?ascbin=8&maxaln=10&seltype=2&uid=pfam02624) |
| sugar phosphate isomerase/epimerase | [**WP_003597811.1**](https://www.ncbi.nlm.nih.gov/protein/WP_003597811.1) | Xylose isomerase-like TIM barrel | [**pfam01261**](https://www.ncbi.nlm.nih.gov/Structure/cdd/cddsrv.cgi?ascbin=8&maxaln=10&seltype=2&uid=pfam01261) |
| Glycosyltransferase family 4 protein | [**WP_003597812.1**](https://www.ncbi.nlm.nih.gov/protein/WP_003597812.1) | Phosphatidyl-myo-inositol mannosyltransferase | [**cd03801**](https://www.ncbi.nlm.nih.gov/Structure/cdd/cddsrv.cgi?ascbin=8&maxaln=10&seltype=2&uid=cd03801) |
| PIG-L family deacetylase | [**WP_012753103.1**](https://www.ncbi.nlm.nih.gov/protein/WP_012753103.1) | GlcNAc-PI de-N-acetylase | [**pfam02585**](https://www.ncbi.nlm.nih.gov/Structure/cdd/cddsrv.cgi?ascbin=8&maxaln=10&seltype=2&uid=pfam02585) |
| Uracil-DNA glycosylase | [**WP_012753104.1**](https://www.ncbi.nlm.nih.gov/protein/WP_012753104.1) | Uncharacterized family of the uracil-DNA glycosylase superfamily | [**cd10035**](https://www.ncbi.nlm.nih.gov/Structure/cdd/cddsrv.cgi?ascbin=8&maxaln=10&seltype=2&uid=cd10035) |
| Lysylphosphatidylglycerol synthase domain-containing protein | [**WP_003597816.1**](https://www.ncbi.nlm.nih.gov/protein/WP_003597816.1) | bifunctional lysylphosphatidylglycerol flippase/synthetase MprF | [**cl41273**](https://www.ncbi.nlm.nih.gov/Structure/cdd/cddsrv.cgi?ascbin=8&maxaln=10&seltype=2&uid=cl41273) |
| PhoR-like sensor histidine kinase | [**WP_012753105.1**](https://www.ncbi.nlm.nih.gov/protein/WP_012753105.1) | PhoR_proteo super family | [**cl37197**](https://www.ncbi.nlm.nih.gov/Structure/cdd/cddsrv.cgi?ascbin=8&maxaln=10&seltype=2&uid=cl37197) |
| **CopA1 heavy metal translocating P-type ATPase** | [**WP_012753106.1**](https://www.ncbi.nlm.nih.gov/protein/WP_012753106.1) | P-type_ATPase_Cu-like | [**cd02094**](https://www.ncbi.nlm.nih.gov/Structure/cdd/cddsrv.cgi?ascbin=8&maxaln=10&seltype=2&uid=cd02094) |
| **Downstream neighboring gene product** | **Accession** | **NCBI Conserved domains** | **Accession** |
| Cu(I)-responsive transcriptional regulator | [**WP_003605161.1**](https://www.ncbi.nlm.nih.gov/protein/WP_003605161.1) | Helix-Turn-Helix DNA binding domain of CueR-like transcription regulators | [**cd01108**](https://www.ncbi.nlm.nih.gov/Structure/cdd/cddsrv.cgi?ascbin=8&maxaln=10&seltype=2&uid=cd01108) |
| M20 family metallopeptidase | [**WP_003605159.1**](https://www.ncbi.nlm.nih.gov/protein/WP_003605159.1) | Zn_peptidase_like superfamily | [**cd05666**](https://www.ncbi.nlm.nih.gov/Structure/cdd/cddsrv.cgi?ascbin=8&maxaln=10&seltype=2&uid=cd05666) |
| Chemotaxis protein | [**WP_082222831.1**](https://www.ncbi.nlm.nih.gov/protein/WP_082222831.1?report=genpept) | No conserved domains |  |
| Acyltransferase | [**WP_043707950.1**](https://www.ncbi.nlm.nih.gov/protein/WP_043707950.1) | Acyltransferase and SGNH-hydrolase domains | [**COG1835**](https://www.ncbi.nlm.nih.gov/Structure/cdd/cddsrv.cgi?ascbin=8&maxaln=10&seltype=2&uid=COG1835) |
| Hypothetical protein | [**WP_244426788.1**](https://www.ncbi.nlm.nih.gov/protein/WP_244426788.1) | No conserved domains |  |
| tRNA pseudouridine(55) synthase TruB | [**WP_003605151.1**](https://www.ncbi.nlm.nih.gov/protein/WP_003605151.1) | tRNA pseudouridine synthase B | [**PRK05389**](https://www.ncbi.nlm.nih.gov/Structure/cdd/cddsrv.cgi?ascbin=8&maxaln=10&seltype=2&uid=PRK05389) |
| 30S ribosome-binding factor RbfA | [**WP_003605149.1**](https://www.ncbi.nlm.nih.gov/protein/WP_003605149.1) | 30S ribosome-binding factor RbfA | [**PRK00521**](https://www.ncbi.nlm.nih.gov/Structure/cdd/cddsrv.cgi?ascbin=8&maxaln=10&seltype=2&uid=PRK00521) |
| Translation initiation factor IF-2 | [**WP_043766249.1**](https://www.ncbi.nlm.nih.gov/protein/WP_043766249.1) | Translation initiation factor IF-2 | [**PRK05306**](https://www.ncbi.nlm.nih.gov/Structure/cdd/cddsrv.cgi?ascbin=8&maxaln=10&seltype=2&uid=PRK05306) |
| RNA-binding protein | [**WP_012753112.1**](https://www.ncbi.nlm.nih.gov/protein/WP_012753112.1) | RNA-binding protein | [**cl35778**](https://www.ncbi.nlm.nih.gov/Structure/cdd/cddsrv.cgi?ascbin=8&maxaln=10&seltype=2&uid=cl35778) |
| Transcription termination factor NusA | [**WP_003598934.1**](https://www.ncbi.nlm.nih.gov/protein/WP_003598934.1) | Transcription elongation factor NusA | [**PRK09202**](https://www.ncbi.nlm.nih.gov/Structure/cdd/cddsrv.cgi?ascbin=8&maxaln=10&seltype=2&uid=PRK09202) |
| Ribosome maturation factor RimP | [**WP_012753113.1**](https://www.ncbi.nlm.nih.gov/protein/WP_012753113.1) | Ribosome maturation factor RimP | [**PRK00092**](https://www.ncbi.nlm.nih.gov/Structure/cdd/cddsrv.cgi?ascbin=8&maxaln=10&seltype=2&uid=PRK00092) |
| CatB-related O-acetyltransferase | [**WP_003598937.1**](https://www.ncbi.nlm.nih.gov/protein/WP_003598937.1) | Xenobiotic acyltransferase | [**cd03349**](https://www.ncbi.nlm.nih.gov/Structure/cdd/cddsrv.cgi?ascbin=8&maxaln=10&seltype=2&uid=cd03349) |
| Aspartate-semialdehyde dehydrogenase | [**WP_012254075.1**](https://www.ncbi.nlm.nih.gov/protein/WP_012254075.1) | Aspartate-semialdehyde dehydrogenase | [**PRK14874**](https://www.ncbi.nlm.nih.gov/Structure/cdd/cddsrv.cgi?ascbin=8&maxaln=10&seltype=2&uid=PRK14874) |
| Hypothetical protein | [**WP_003598944.1**](https://www.ncbi.nlm.nih.gov/protein/WP_003598944.1) | No conserved domains |  |
| Hypothetical protein | [**WP_003598945.1**](https://www.ncbi.nlm.nih.gov/protein/WP_003598945.1) | No conserved domains |  |
|  |  |  |  |
| ***copA2* (Fig. 5)** | | | |
| **Upstream neighboring gene product** | **Accession** | **NCBI Conserved domains** | **Accession** |
| Relaxase domain-containing protein | [**WP_012752679.1**](https://www.ncbi.nlm.nih.gov/protein/WP_012752679.1) | TrwC relaxase super family | [**cl19905**](https://www.ncbi.nlm.nih.gov/Structure/cdd/cddsrv.cgi?ascbin=8&maxaln=10&seltype=2&uid=cl19905) |
| Hypothetical protein | [**WP_012752681.1**](https://www.ncbi.nlm.nih.gov/protein/WP_012752681.1) | No conserved domains |  |
| Hypothetical protein | [**WP_012752682.1**](https://www.ncbi.nlm.nih.gov/protein/WP_012752682.1) | No conserved domains |  |
| Hypothetical protein | [**WP_012752683.1**](https://www.ncbi.nlm.nih.gov/protein/WP_012752683.1) | No conserved domains |  |
| Type IV secretory system conjugative DNA transfer family protein | [**WP_012752684.1**](https://www.ncbi.nlm.nih.gov/protein/WP_012752684.1) | Type IV secretory system Conjugative DNA transfer | [**cl29730**](https://www.ncbi.nlm.nih.gov/Structure/cdd/cddsrv.cgi?ascbin=8&maxaln=10&seltype=2&uid=cl29730) |
| Hypothetical protein | [**WP_043766046.1**](https://www.ncbi.nlm.nih.gov/protein/WP_043766046.1) | No conserved domains |  |
| IS3 family transposase | [**WP_085985459.1**](https://www.ncbi.nlm.nih.gov/protein/WP_085985459.1) | IS3 family transposase; | [**NF033516**](https://www.ncbi.nlm.nih.gov/Structure/cdd/cddsrv.cgi?ascbin=8&maxaln=10&seltype=2&uid=NF033516) |
| Zinc-like metallopeptidase domain-containing protein | [**WP_043766049.1**](https://www.ncbi.nlm.nih.gov/protein/WP_043766049.1) | Antirestriction protein ArdC | [**cl39143**](https://www.ncbi.nlm.nih.gov/Structure/cdd/cddsrv.cgi?ascbin=8&maxaln=10&seltype=2&uid=cl39143) |
| Hypothetical protein | [**WP_043766051.1**](https://www.ncbi.nlm.nih.gov/protein/WP_043766051.1) | No conserved domains |  |
| PIN domain-containing protein | [**WP_043766056.1**](https://www.ncbi.nlm.nih.gov/protein/WP_043766056.1) | VapC-like PIN domain family Type II toxin-antitoxin system | [**cl28905**](https://www.ncbi.nlm.nih.gov/Structure/cdd/cddsrv.cgi?ascbin=8&maxaln=10&seltype=2&uid=cl28905) |
| Type II toxin-antitoxin system Phd/YefM family antitoxin | [**WP_043766059.1**](https://www.ncbi.nlm.nih.gov/protein/WP_043766059.1) | Phd/YefM antitoxin family Type II toxin-antitoxin system | [**TIGR01552**](https://www.ncbi.nlm.nih.gov/Structure/cdd/cddsrv.cgi?ascbin=8&maxaln=10&seltype=2&uid=TIGR01552) |
| Hypothetical protein | [**WP_043766062.1**](https://www.ncbi.nlm.nih.gov/protein/WP_043766062.1) | No conserved domains |  |
| Recombinase family protein | [**WP_043766064.1**](https://www.ncbi.nlm.nih.gov/protein/WP_043766064.1) | Serine Recombinase (SR) family, Resolvase and Invertase subfamily | [**cd03768**](https://www.ncbi.nlm.nih.gov/Structure/cdd/cddsrv.cgi?ascbin=8&maxaln=10&seltype=2&uid=cd03768) |
| Isoprenylcysteine carboxylmethyltransferase family protein | [**WP_012752685.1**](https://www.ncbi.nlm.nih.gov/protein/WP_012752685.1) | Protein-S-isoprenylcysteine O-methyltransferase Ste14 | [**COG2020**](https://www.ncbi.nlm.nih.gov/Structure/cdd/cddsrv.cgi?ascbin=8&maxaln=10&seltype=2&uid=COG2020) |
| **CopA2 heavy metal translocating P-type ATPase** | [**WP_158022369.1**](https://www.ncbi.nlm.nih.gov/protein/WP_158022369.1) | P-type_ATPase_Cu-like | [**cd02094**](https://www.ncbi.nlm.nih.gov/Structure/cdd/cddsrv.cgi?ascbin=8&maxaln=10&seltype=2&uid=cd02094) |
| **Downstream neighboring gene product** | **Accession** | **NCBI Conserved domains** | **Accession** |
| DUF3141 domain-containing protein | [**WP_244426907.1**](https://www.ncbi.nlm.nih.gov/protein/WP_244426907.1) | Protein of unknown function (DUF3141) | [**cl26471**](https://www.ncbi.nlm.nih.gov/Structure/cdd/cddsrv.cgi?ascbin=8&maxaln=10&seltype=2&uid=cl26471) |
| Acetate/propionate family kinase | [**WP_012752688.1**](https://www.ncbi.nlm.nih.gov/protein/WP_012752688.1) | NBD_sugar-kinase_HSP70_actin super family | [**cl17037**](https://www.ncbi.nlm.nih.gov/Structure/cdd/cddsrv.cgi?ascbin=8&maxaln=10&seltype=2&uid=cl17037) |
| Bifunctional enoyl-CoA hydratase/phosphate acetyltransferase | [**WP_012752689.1**](https://www.ncbi.nlm.nih.gov/protein/WP_012752689.1) | Bifunctional enoyl-CoA hydratase/phosphate acetyltransferase | [**cl28521**](https://www.ncbi.nlm.nih.gov/Structure/cdd/cddsrv.cgi?ascbin=8&maxaln=10&seltype=2&uid=cl28521) |
| TetR/AcrR family transcriptional regulator | [**WP_012752690.1**](https://www.ncbi.nlm.nih.gov/protein/WP_012752690.1) | Bacterial transcriptional repressor | [**pfam16859**](https://www.ncbi.nlm.nih.gov/Structure/cdd/cddsrv.cgi?ascbin=8&maxaln=10&seltype=2&uid=pfam16859) |
| IS3 family transposase | [**WP_085985459.1**](https://www.ncbi.nlm.nih.gov/protein/WP_085985459.1) | IS3 family transposase | [**NF033516**](https://www.ncbi.nlm.nih.gov/Structure/cdd/cddsrv.cgi?ascbin=8&maxaln=10&seltype=2&uid=NF033516) |
|  |  |  |  |
